# Higher fasting blood glucose was associated with worse in‐hospital clinical outcomes in patients with primary intracerebral hemorrhage: From a large‐scale nationwide longitudinal registry

**DOI:** 10.1111/cns.13972

**Published:** 2022-09-24

**Authors:** Guangshuo Li, Shang Wang, Yunyun Xiong, Hongqiu Gu, Yingyu Jiang, Xin Yang, Chunjuan Wang, Chuanying Wang, Zixiao Li, Xingquan Zhao

**Affiliations:** ^1^ Department of Neurology, Beijing Tiantan Hospital Capital Medical University Beijing China; ^2^ Neurocardiology Center, Department of Neurology, Beijing Tiantan Hospital Capital Medical University Beijing China; ^3^ Chinese Institute for Brain Research Beijing China; ^4^ China National Clinical Research Center for Neurological Diseases Beijing China; ^5^ National Center for Healthcare Quality Management in Neurological Diseases Beijing China

**Keywords:** cerebral hemorrhage, glucose, mortality, outcome

## Abstract

**Introduction:**

Studies that investigated the relationship between fasting blood glucose (FBG) and intracerebral hemorrhage (ICH) outcomes were insufficient.

**Aim:**

We aimed to investigate the association between FBG level and in‐hospital clinical outcomes in patients with primary ICH.

**Results:**

A total of 34,507 patients were enrolled in the final study. Compared with the reference group, the ≥6.1 and <7 mmol/L group showed nonsignificant higher in‐hospital mortality (adjusted odds ratio [OR] 1.20, 95% confidence interval [CI] 0.69–2.11, *p* = 0.52), and a significant higher proportion of intracranial hematoma evacuation (adjusted OR 1.56, 95% CI 1.26–1.92, *p* < 0.001). The ≥7 mmol/L group showed both significant higher in‐hospital mortality (adjusted OR 2.08, 95% CI 1.42–3.04, *p* = 0.52) and a significant higher proportion of intracranial hematoma evacuation (adjusted OR 2.09, 95% CI 1.78–2.47, *p* < 0.001).

**Conclusion:**

Higher FBG level was correlated with both higher mortality and proportion of evacuation of intracranial hematoma.

## Graphical abstract

Studies that investigated the relationship between fasting blood glucose (FBG) and intracerebral hemorrhage (ICH) outcomes were insufficient. We aimed to investigate the association between FBG level and in‐hospital clinical outcomes in patients with primary ICH. Higher FBG level was correlated with both higher mortality and proportion of evacuation of intracranial hematoma.

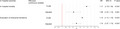

## 1 INTRODUCTION

Primary intracerebral hemorrhage (ICH) shows increased percentage of disability and mortality compared with acute ischemic stroke. 54% of the patients failed to survive in the first year after the occurrence of ICH.^1^ Hyperglycemia after the occurrence of ischemic stroke is a predictor of poor clinical outcomes and guidelines for the early management of patients with acute ischemic stroke recommended to avoid hyperglycemia to improve clinical outcomes.^2^ Some studies investigated the relationship between admission random glucose and ICH outcomes.^3^, ^4^, ^5^, ^6^, ^7^ It remained uncertain whether hyperglycemia was correlated with poor outcomes in ICH. After ICH occurred, high blood glucose may further aggravate brain edema, blood–brain barrier destruction and neuronal apoptosis, resulting in poor clinical outcomes.^8^, ^9^ However, random plasma glucose might not be an optimal glucose parameter considering its great intraindividual variation.^10^ Besides, some glucose disorders may occur at 2–4 days after ICH.^11^ Fasting blood glucose (FBG) might be more appropriate, but it remained uncertain whether FBG was associated with ICH outcomes.

In this study, we aimed to investigate the relationship between FBG level and in‐hospital mortality in ICH patients. Considering that FBG may be elevated for the stress response, we further conducted subgroup analyses between patients with a history of diabetes mellitus or not; patients with a medication history of antidiabetic agents or not; and patients with a HbA1c ≥7.0% or <7.0%.

## 2 METHOD

### 2.1 Study design and participants

The data was retrieved from a multicenter database, the China Stroke Center Alliance (CSCA). The CSCA was a national registry cohort that enrolled patients with acute stroke/transient ischemic attack from 1476 hospitals in China.^12^ This CSCA program provided a national platform to collect electronic data and improve stroke care in China, similar to the American Heart Association's Get With the Guidelines‐Stroke (GWTG‐Stroke) program in America.^13^ All of the secondary and tertiary hospitals in China were permitted to upload clinical data of stroke patients. Between 2015 and 2019, 1,006,798 patients diagnosed with stroke or TIA confirmed by cranial CT or MRI were recruited in the CSCA cohort consecutively, of whom 85,705 were ICH patients. Patients were enrolled into the current study if aged over 50 years and with a diagnosis of spontaneous ICH. Patients were excluded if the clinical or laboratory information, particularly GCS score and FBG results, were incomplete.

### 2.2 Baseline characteristics and data collection

Data collection was conducted at each subcenter. We removed all identifiers on each data before statistical analysis and to protect the privacy and confidential of the recruited patients, the data was only accessible with the permission from the China National Clinical Research Center for Neurological Diseases. Baseline information of the enrolled patients was uploaded and saved in a web‐based patient data collection and management tool (Medicine Innovation Research Center, Beijing, China). FBG samples were collected in the second morning of admission at each subcenter. The enrolled patients were categorized into three groups according to FBG levels^10^, ^14^, ^15^: (1) 3.9–6.1 mmol/L (70–110 mg/dl); (2) ≥6.1 and <7 mmol/L (110–125 mg/dl) (3) ≥7 mmol/L (125 mg/dl). We determined the normal FBG level 3.9–6.1 mmol/L (70–110 mg/dl) as the reference group. Other baseline information including age, sex, Glasgow coma scale (GCS) score, medical history (hypertension, diabetes mellitus, atrial fibrillation, dyslipidemia, prior stroke), smoking, drinking, and laboratory measures. Moreover, the CSCA was designed to collect in‐hospital information of the enrolled patients without follow‐up data after discharge.

### 2.3 Outcome assessment

We defined the primary outcome of our study as in‐hospital mortality. Secondary study outcome was defined as the percentage of the evacuation of intracerebral hematoma to estimate the percentage of hematoma occupying or hematoma expansion, considering the information on the percentage of hematoma occupying or hematoma expansion was absent in the original data collection.

### 2.4 Subgroup analyses

The first subgroup analysis was conducted in patients with or without a history of diabetes mellitus; the second subgroup analysis was conducted in patients with or without a medication history of antidiabetic agents or not; the third subgroup analysis was conducted in patients with a HbA1c ≥7.0% or <7.0%, to determine the impact of different FBG levels on the outcomes.

### 2.5 Statistical analysis

Continuous variables were expressed as mean ± SD for normally distributed data and median (interquartile range) for skewed distributed data. The data were tested for a normal distribution using the Kolmogorov–Smirnov test. Categorical variables were expressed as number (percentage). In univariate analyses, normally distributed data were compared using the ANOVA analyses and non‐normally distributed data were compared using Kruskal–Wallis tests. Categorical variables were compared using χ2 tests or trend tests, as appropriate. Multicollinearity was assessed via variation inflation factors (VIF), and VIF ≥5.0 was considered significant. Multiple logistic analyses were also performed. All covariates with a *p* value ≤ 0.1 (confounders) in the comparison of baseline characteristics and different FBG levels were entered into the multiple logistic regression. Logistic regression models were also used to test interaction effect. We determined the normal FBG level 3.9–6.1 mmol/L (70–110 mg/dl) as the reference group. A two‐sided *p* value < 0.05 was considered to be statistically significant. All analyses were performed with SAS software version 9.4 (SAS Institute, Inc.).

## 3 RESULTS

**FIGURE 1:**
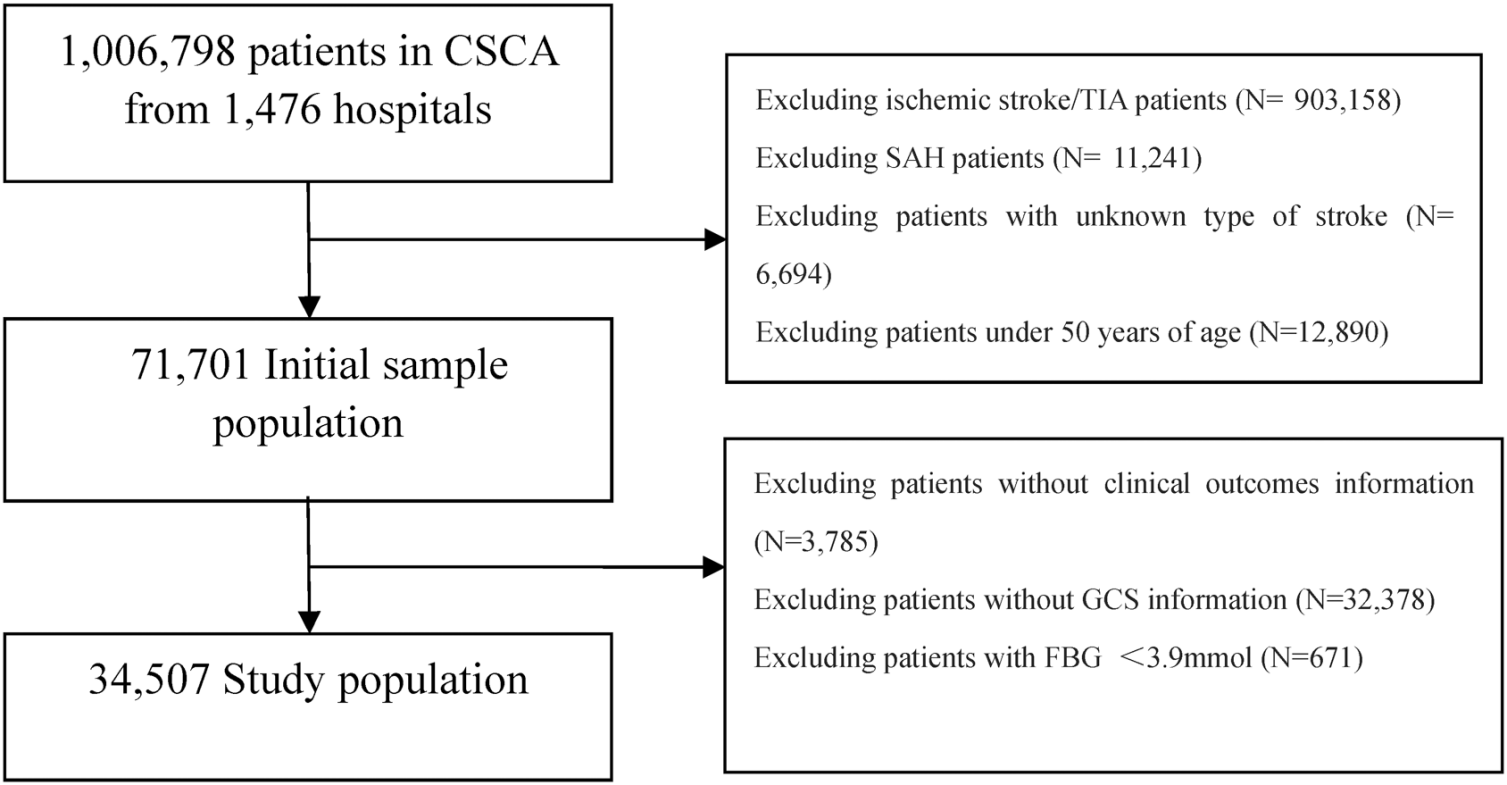
Flow chart

A total of 34,507 patients were enrolled in the final study. (Figure 1) The included patients had a mean age of 66.3 ± 10.3 years, and 21,001 (60.9%) of them were men. The 3.9–6.1 mmol/L group had 18,722 (54.3%) patients, the ≥6.1 and <7 mmol/L group had 4669 (13.5%) patients and the ≥7 mmol/L group had 11,116 (32.2%). Comparison of baseline characteristics is summarized in Table 1. Compared with the 3.9–6.1 mmol/L and ≥6.1 and <7 mmol/L group, the ≥7 mmol/L group had a higher proportion of men (6242 [56.2%], *p* < 0.001), history of hypertension (8506 [76.5%], *p* < 0.001), history of DM (2615 [23.5%], *p* < 0.001), history of dyslipidemia (536 [4.8%], *p* < 0.001), history of atrial fibrillation (216 [1.9%], *p* < 0.01), history of myocardial infarction (152 [1.4%], *p* < 0.001), history of ischemic stroke (1648 [14.8%], *p* < 0.001), history of intracerebral hemorrhage (2076 [18.7%], *p* < 0.001). Both the fasting plasma glucose and HbA1C level were higher in the ≥7 mmol/L group compared with those in the other two groups (median FBG: 8.5 mmol/L, *p* < 0.001; median HbA1C: 6.7%, *p* < 0.001). The ≥7 mmol/L group also had higher systolic BP (172.4 ± 30.2 mmHg, *p* < 0.001) and diastolic BP (96.9 ± 17.4 mmHg, *p* < 0.001). (Table 1) No significant collinearity or interaction was observed between baseline characteristics.

**TABLE 1:** Demographic and clinical characteristics between different FBG levels

	Total (*N* = 34,507)	3.9–6.1 mmol/L group (*N* = 18,722)	≥6.1 and <7 mmol/L group (*N* = 4669)	≥7 mmol/L group (*N* = 11,116)	*p* value
Demographic					
Age (Mean ± SD, years)	66.3 ± 10.3	66.2 ± 10.3	66.7 ± 10.3	66.4 ± 10.3	<0.01
Male (*n*, %)	21,001 (60.9)	11,970 (63.9)	2789 (59.7)	6242 (56.2)	<0.001
GCS at admission (Mean ± SD)	11.4 ± 4.1	12.3 ± 3.7	11.3 ± 4.0	10.0 ± 4.3	<0.001
Medical history					
Hypertension (*n*, %)	25,346 (73.5)	13,389 (71.5)	3451 (73.9)	8506 (76.5)	<0.001
Diabetes mellitus (*n*, %)	3488 (10.1)	579 (3.1)	294 (6.3)	2615 (23.5)	<0.001
Dyslipidemia (*n*, %)	1280 (3.7)	592 (3.2)	152 (3.3)	536 (4.8)	<0.001
Atrial fibrillation (*n*, %)	552 (1.6)	266 (1.4)	70 (1.5)	216 (1.9)	<0.01
Myocardial infarction (*n*, %)	355 (1.0)	162 (0.9)	41 (0.9)	152 (1.4)	<0.001
Ischemic stroke (*n*, %)	4600 (13.3)	2349 (12.5)	603 (12.9)	1648 (14.8)	<0.001
Intracerebral hemorrhage (*n*, %)	6036 (17.5)	3126 (16.7)	834 (17.9)	2076 (18.7)	<0.001
Smoking (*n*, %)	6283 (18.2)	3729 (19.9)	805 (17.2)	1749 (15.7)	<0.001
Alcoholism (*n*, %)	8028 (23.3)	4565 (24.4)	1092 (23.4)	2371 (21.3)	<0.001
Antiplatelet agents (*n*, %)	2467 (7.1)	1293 (6.9)	304 (6.5)	870 (7.8)	0.002
Anticoagulant agents (*n*, %)	689 (2.0)	343 (1.8)	86 (1.8)	260 (2.3)	0.007
Antihypertensive agents (*n*, %)	17,180 (49.8)	8959 (47.9)	2340 (50.1)	5881 (52.9)	<0.0001
Antidiabetic agents (*n*, %)	2601 (7.5)	411 (2.2)	211 (4.5)	1979 (17.8)	<0.0001
Laboratory Findings					
LDL‐C (Mean ± SD, mmol/L)	2.8 ± 1.6	2.8 ± 1.5	2.8 ± 1.5	2.9 ± 1.8	<0.001
HDL‐C (Mean ± SD, mmol/L)	1.1 ± 0.9	1.2 ± 1.0	1.1 ± 0.9	1.1 ± 0.9	0.5
Total cholesterol (Mean ± SD, mmol/L)	1.0 ± 1.9	1.0 ± 1.9	1.0 ± 1.9	1.1 ± 2.0	<0.001
Triglyceride (Mean ± SD, mmol/L)	0.4 ± 1.2	0.4 ± 1.1	0.5 ± 1.3	0.5 ± 1.3	<0.001
Fasting plasma glucose (Mean ± SD, mmol/L)	6.0 (5.2–7.4)	5.3 (4.9–5.7)	6.5 (6.3–6.7)	8.5 (7.5–10.3)	<0.001
HbAlc (Mean ± SD, %)	6.0 ± 1.7	5.5 ± 1.2	5.8 ± 1.3	6.7 ± 2.2	<0.001
Systolic blood pressure (Mean ± SD, mmHg)	166.5 ± 28.1	162.7 ± 26.2	167.7 ± 27.4	172.4 ± 30.2	<0.001
Diastolic blood pressure (Mean ± SD, mmHg)	94.9 ± 16.3	93.6 ± 15.5	95.3 ± 16.3	96.9 ± 17.4	<0.001
In‐hospital mortality	1075 (3.1)	257 (1.4)	111 (2.4)	707 (6.4)	<0.001
Percentage of the evacuation of intracranial hematoma	5021 (14.6)	1667 (8.9)	737 (15.8)	2617 (23.5)	<0.001

### 3.1 Fasting glucose level and outcomes

The in‐hospital mortality was 3.1% (1075/34,507) of the included patients, and a total of 5021 (14.6%) patients underwent evacuation of intracranial hematoma before discharge. Compared with the 3.9–6.1 mmol/L and ≥6.1 and <7 mmol/L group, the ≥7 mmol/L group had a higher in‐hospital mortality (6.4%, *p* < 0.001) and proportion of the evacuation of intracranial hematoma (23.5%, *p* < 0.001). (Table 1).

**FIGURE 2:**
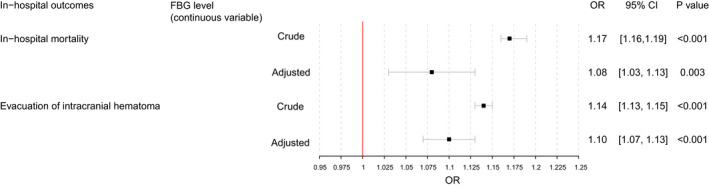
Forest plots of in‐hospital outcomes. FBG, fasting blood glucose. Adjusted for age, male, GCS score, medical history, LDL‐c, total cholesterol, triglyceride, HbA1c, systolic blood pressure and diastolic blood pressure

**FIGURE 3:**
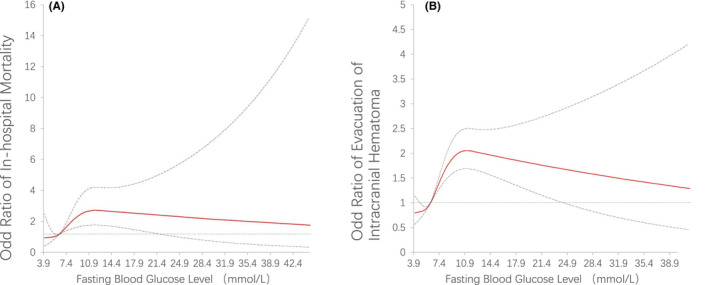
Restricted cubic splines to delineate the relationship between FBG and adjusted OR for (A) in‐hospital mortality, and (B) evacuation of intracranial hematoma. Adjusted for age, male, GCS score, medical history, LDL‐c, total cholesterol, triglyceride, HbA1c, systolic blood pressure and diastolic blood pressure

Logistics regression analyses showed that, compared with the 3.9–6.1 mmol/L group, both the ≥6.1 and <7 mmol/L and ≥7 mmol/L groups had a higher proportion of in‐hospital mortality (≥6.1 and <7 mmol/L group: crude OR, 1.75, 95% CI 1.40–2.19, *p* < 0.001; ≥7 mmol/L group: crude OR, 4.88, 95% CI 4.22–5.64, *p* < 0.001). This association remained significant after adjusting for confounding factors (≥6.1 and <7 mmol/L group: adjusted OR, 1.20, 95% CI 0.69–2.11, *p* = 0.52; ≥7 mmol/L group: adjusted OR, 2.08, 95% CI: 1.42–3.04, *p* < 0.001). Similarly, compared with the 3.9–6.1 mmol/L group, both the ≥6.1 and <7 mmol/L and ≥7 mmol/L groups had a higher risk for evacuation of intracranial hematoma (≥6.1 and <7 mmol/L group: crude OR, 1.92, 95% CI 1.75–2.11, *p* < 0.001; ≥7 mmol/L group: crude OR, 3.15, 95% CI 2.95–3.37, *p* < 0.001). Adjusted models also showed that both the ≥6.1 and <7 mmol/L and ≥7 mmol/L groups had a higher risk for evacuation of intracranial hematoma (≥6.1 and <7 mmol/L group: adjusted OR, 1.56, 95% CI 1.26–1.92, *p* < 0.001; ≥7 mmol/L group: adjusted OR, 2.09, 95% CI 1.78–2.47, *p* < 0.001). Table 2 As a continuous variable, higher FBG level showed a higher likelihood of in‐hospital mortality and undergoing evacuation of intracranial hematoma, both in the univariate (crude OR for in‐hospital mortality, 1.17, 95% CI 1.16–1.19, *p* < 0.001; crude OR for evacuation of intracranial hematoma, 1.14, 95% CI 1.13–1.15, *p* < 0.001) and multivariable (adjusted OR for in‐hospital mortality, 1.08, 95% CI 1.03–1.13, *p* < 0.001; adjusted OR for evacuation of intracranial hematoma, 1.10, 95% CI 1.07–1.13, *p* < 0.001) models. Figure 2 RCS showed that a J‐shaped curve between FBG level and in‐hospital clinical outcomes, indicating that FBG level higher than 6 mmol/L was significantly correlated with worse clinical outcomes. Moreover, the OR value was close to 1, and the lower bound of 95% CI crossed 1 when FBG level was greater than 20, indicating the correlation between FBG and outcomes was nonstatistical. (Figure 3).

**TABLE 2:** Logistics regression analyses of in‐hospital outcomes

In‐hospital outcomes	FBG level		OR	95% CI	*p* value
In‐hospital mortality	3.9–6.1 mmol/L		reference	reference	reference
	≥6.1 and <7 mmol/L	Crude	1.75	1.40–2.19	<0.001
		Adjusted	1.20	0.69–2.11	0.52
	≥7 mmol/L	Crude	4.88	4.22–5.64	<0.001
		Adjusted	2.08	1.42–3.04	<0.001
Evacuation of intracranial hematoma	3.9–6.1 mmol/L		reference	reference	reference
	≥6.1 and <7 mmol/L	Crude	1.92	1.75–2.11	<0.001
		Adjusted	1.56	1.26–1.92	<0.001
	≥7 mmol/L	Crude	3.15	2.95–3.37	<0.001
		Adjusted	2.09	1.78–2.47	<0.001

*Note*: Adjusted for age, male, GCS score, medical history, LDL‐c, total cholesterol, triglyceride, HbA1c, systolic blood pressure and diastolic blood pressure.

### 3.2 Subgroup analyses

In patients without history of DM, compared with the 3.9–6.1 mmol/L group, the ≥6.1 and <7 mmol/L group had a higher risk for in‐hospital mortality (crude OR, 4.79, 95% CI 4.11–5.58, *p* < 0.001; adjusted OR 2.04, 95% CI 1.37–3.03, *p* < 0.001). The ≥6.1 and <7 mmol/L group had a trend for increasing the risk for in‐hospital mortality, but the association was nonsignificant after adjusting for confounders (crude OR, 1.74, 95% CI 1.38–2.19, *p* < 0.001; adjusted OR 1.10, 95% CI 0.61–1.99, *p* = 0.75). The ≥7 mmol/L and ≥6.1 and <7 mmol/L groups in patients without history of DM also had higher risk for evacuation of intracranial hematoma, compared with those in the 3.9–6.1 mmol/L group (≥7 mmol/L group: crude OR, 3.50, 95% CI 3.26–3.75, *p* < 0.001; adjusted OR 2.16, 95% CI 1.82–2.56, *p* < 0.001; ≥6.1 and <7 mmol/L group: crude OR, 1.99, 95% CI 1.81–2.19, *p* < 0.001; adjusted OR 1.59, 95% CI 1.28–1.97, *p* < 0.001). (Table S1).

In patients without medication history of antidiabetic agents, compared with the 3.9–6.1 mmol/L group, ≥7 mmol/L groups had a higher risk for in‐hospital mortality (crude OR, 4.91, 95% CI 4.22–5.71, *p* < 0.001; adjusted OR 2.09, 95% CI 1.41–3.08, *p* < 0.001). The ≥6.1 and <7 mmol/L group had a similar intendency but the association with in‐hospital mortality was nonsignificant after adjusting for confounders (crude OR, 1.75, 95% CI 1.39–2.20, *p* < 0.001; adjusted OR 1.10, 95% CI 0.61–1.99, *p* = 0.74). The ≥7 mmol/L and ≥6.1 and <7 mmol/L groups in patients without history of DM also had higher risk for evacuation of intracranial hematoma, compared with those in the 3.9–6.1 mmol/L group (≥7 mmol/L group: crude OR, 3.39, 95% CI 3.17–3.64, *p* < 0.001; adjusted OR 2.11, 95% CI 1.79–2.49, *p* < 0.001; ≥6.1 and <7 mmol/L: crude OR, 1.97, 95% CI 1.79–2.16, *p* < 0.001; adjusted OR 1.58, 95% CI 1.28–1.96, *p* < 0.001). (Table S2).

In patients with a HbA1C level <7, compared with the 3.9–6.1 mmol/L group, the ≥7 mmol/L group had a higher risk for in‐hospital mortality (crude OR, 5.12, 95% CI 4.39–5.97, *p* < 0.001; adjusted OR 2.11, 95% CI 1.42–3.12, *p* < 0.001). The ≥6.1 and <7 mmol/L group had a trend for in‐hospital mortality but the correlation was nonsignificant after adjusting for confounders (crude OR, 1.81, 95% CI 1.44–2.28, *p* < 0.01; adjusted OR 1.29, 95% CI 0.73–2.28, *p* = 0.38). The ≥7 mmol/L and ≥6.1 and <7 mmol/L groups in patients without history of DM also had higher risk for evacuation of intracranial hematoma, compared with those in the 3.9–6.1 mmol/L group (≥7 mmol/L group: crude OR, 3.49, 95% CI 3.25–3.75, *p* < 0.001; adjusted OR 2.01, 95% CI 1.69–2.38, *p* < 0.001; ≥6.1 and <7 mmol/L group: crude OR, 1.95, 95% CI 1.77–2.15, *p* < 0.001; adjusted OR 1.48, 95% CI 1.19–1.84, *p* < 0.001). (Table S3).

## 4 DISCUSSION

The current study found that a higher FBG level was associated with a higher risk for in‐hospital mortality and intracranial hematoma evacuation in ICH patients. With a large study population of patients with ICH, our study confirmed the close correlation between FBG level and in‐hospital clinical outcomes in the entire study sample and different subgroups.

A posthoc study of the Intensive Blood Pressure Reduction in Acute Cerebral Hemorrhage Trial (INTERACT2) confirmed that both hyperglycemia and DM were predictors of poor clinical outcomes (mRS 3–6) at 90 days of ICH.^16^ In accordance with the post‐hoc study of the INTERACT2, we also observed that a higher FBG level was related with higher in‐hospital mortality in ICH patients. Besides, similar with ≥7 mmol/L group, our study showed that ≥6.1 and <7 mmol/L group might also have an elevated risk of death or evacuation of intracranial hematoma. Patients with impaired fasting glucose may be ignored considering milder increment of glucose compared with the moderate or severe increment of DM patients. Another large cohort also showed significantly increased risk of death and poor clinical outcomes (mRS 2–6) at 1 year in ICH patients with impaired glucose regulation compared with those in ICH patients with normal glucose level.^17^

Subgroup analyses found that higher FBG was significantly correlated with higher in‐hospital mortality and evacuation rates of intracranial hematoma in patients without DM history or medication history of antidiabetic agents, as well as in patients with a HbA1C level <7. Our findings are consistent with a large observation analysis based on the China National Stroke Registry (*n* = 2951), which also showed that elevated admission blood glucose indicated a higher risk of poor outcome in nondiabetics than diabetics with similar glucose levels.^18^ This phenomenon may be due to stress and insulin resistance indicating poor clinical outcomes in ICH patients without history of DM compared with patients with history of DM.^18^

From pathophysiological point of view, post‐ICH hyperglycemia might be initiated through a neuroendocrine stress‐mediated process.^19^ Destruction of BBB enabled brain tissue exposed to excessive levels of glucose and caused cytotoxicity and perihematomal neuronal death due to anaerobic glycolysis,^16^ lactic acid accumulation,^16^ activation of excitory amino acids, and calcium overload.^20^ Hyperglycemia and destruction of BBB might also generate vasogenic brain edema, which may increase the risk of death.^3^, ^8^, ^9^ Lately, intraventricular extension was found to be associated with hyperglycemia.^3^ This linear correlation between the intraventricular extension score and glucose level indicated that intraventricular extension events may be a mediator between hyperglycemia and poor outcomes including death.

Neuro‐immune process was reported to play a vital role in clinical outcomes of ICH. Cheng et al. found that reduced level of programmed death‐ligand 1 (PD‐L1) in the perihematomal area and elevated level of PD‐L1 in the peripheral blood, resulting in immunosuppression and higher risk of pulmonary infection.^21^ Toll‐like receptors 9‐enhanced macrophage/microglial phagocytosis also played a neuroprotective role via the Nrf2/CD204 pathway in ICH patients.^22^ Besides, iron overload, due to erythrolysis extent, could induce secondary injury via aggravated BBB leakage, increased glia and matrix metalloproteinase‐9 accumulation in hypertensive ICH.^23^, ^24^ BBB damage was also found to be associated with augmentation of endothelial Wnt/β‐catenin signaling, and lithium could upregulate this signal pathway to treat BBB breakdown.^25^ Moreover, several clinical factors were associated with outcomes of ICH. Prior statin was associated with reduced proportion of evacuation of intracranial hematoma without increasing mortality during in‐hospitalization.^26^ The cost‐effectiveness ratio of anticoagulant therapy is still uncertain in ICH and more investigations are warranted,^27^, ^28^ as well as the role of platelets and onset time that have been studied in subarachnoid hemorrhage^29^ and ischemic stroke.^30^

Several limitations need to be paid attention to in our study. First, our study was a retrospective design based on an observational cohort and may have had recall and selection bias. Second, the CSCA database was not intended to include the 90 days or long‐term followup information, which makes it impossible for us to analyze the functional status after discharge in these ICH patients. Third, CSCA was a quality improvement program of stroke treatment in the neurology. Hence, the patients admitted into neurosurgery or intensive care unit, particularly the patients with more severe neurological impairment or higher mortality risk, may not be enrolled in the CSCA. The ICH patients in the CSCA may have a higher median GCS score and lower morality rate. Fourth, data collection of hematoma volume and other imaging information (ICH location and IVH extension) was not achievable considering different evaluating methods to compute the hematoma volume in the hospitals participating CSCA program (automatic software, semiautomatic software, or manually). GCS score was collected to reflect the hematoma volume. Although significant differences on median GCS score were observed among study groups, we used multivariable logistics regression analyses to adjust for confounders including GCS score.

## 5 CONCLUSION

Higher FBG level was correlated with higher risk for in‐hospital mortality and intracranial hematoma evacuation.

## CONFLICT OF INTEREST

The authors declare that they have no competing interests.

## AUTHOR CONTRIBUTIONS

X.Z., Z.L., and Y.X. contributed to the conception and design of the study; H.G., Y.J., X.Y., C.W., and C.W. contributed to the acquisition and analysis of data; G.L. contributed to drafting the text and preparing the figures. S.W. contributed to preparing the figures.

## Supporting information


Table S1
Table S2
Table S3

## Data Availability

The data that support the findings of this study are available from the corresponding author upon reasonable request.
