# Supplementary material for: Higher fasting blood glucose was associated with worse in‐hospital clinical outcomes in patients with primary intracerebral hemorrhage: From a large‐scale nationwide longitudinal registry
Source: CNS Neurosci Ther. 2022 Sep 24;28(12):2260–7. doi: 10.1111/cns.13972 (PMC9627374; doi:10.1111/cns.13972)
Supplement: Supplementary file 1 — Table S1 Table S2 Table S3 [file CNS-28-2260-s001.docx]

Supplemental.Table.1 Subgroup analyses (with vs. without DM history) of in–hospital outcomes

|  | In−hospital outcomes | FBG level | OR | 95% CI | P value |
| --- | --- | --- | --- | --- | --- |
| With a history of DM | In−hospital mortality | 3.9–6.1 mmol/L | reference | reference | reference |
|  |  | ≥6.1 and < 7 mmol/L | 4.88 | 0.57-41.58 | 0.15 |
|  |  | ≥7 mmol/L | 3.37 | 0.70-16.18 | 0.13 |
|  | Evacuation of intracranial hematoma | 3.9–6.1 mmol/L | reference | reference | reference |
|  |  | ≥6.1 and < 7 mmol/L | 0.76 | 0.25-2.32 | 0.63 |
|  |  | ≥7 mmol/L | 1.16 | 0.58-2.30 | 0.68 |
| Without a history of DM | In−hospital mortality | 3.9–6.1 mmol/L | reference | reference | reference |
|  |  | ≥6.1 and < 7 mmol/L | 1.01 | 0.61-1.99 | 0.75 |
|  |  | ≥7 mmol/L | 2.04 | 1.37-3.03 | <0.001 |
|  | Evacuation of intracranial hematoma | 3.9–6.1 mmol/L | reference | reference | reference |
|  |  | ≥6.1 and < 7 mmol/L | 1.59 | 1.28-1.97 | <0.001 |
|  |  | ≥7 mmol/L | 2.16 | 1.82-2.56 | <0.001 |

Adjusted for age, male, GCS score, medical history, LDL-c, total cholesterol, triglyceride, HbA1c, systolic blood pressure and diastolic blood pressure.

Supplemental.Table.2 Subgroup analyses (with vs. without antidiabetic therapy) of in–hospital outcomes

|  | In−hospital outcomes | FBG level | OR | 95% CI | P value |
| --- | --- | --- | --- | --- | --- |
| With antidiabetic therapy | In−hospital mortality | 3.9–6.1 mmol/L | reference | reference | reference |
|  |  | ≥6.1 and < 7 mmol/L | 2.16 | 0.24-19.92 | 0.50 |
|  |  | ≥7 mmol/L | 1.38 | 0.26-7.45 | 0.71 |
|  | Evacuation of intracranial hematoma | 3.9–6.1 mmol/L | reference | reference | reference |
|  |  | ≥6.1 and < 7 mmol/L | 0.54 | 0.14-2.15 | 0.38 |
|  |  | ≥7 mmol/L | 0.94 | 0.40-2.19 | 0.88 |
| Without antidiabetic therapy | In−hospital mortality | 3.9–6.1 mmol/L | reference | reference | reference |
|  |  | ≥6.1 and < 7 mmol/L | 1.10 | 0.61-1.99 | 0.74 |
|  |  | ≥7 mmol/L | 2.09 | 1.41-3.08 | <0.001 |
|  | Evacuation of intracranial hematoma | 3.9–6.1 mmol/L | reference | reference | reference |
|  |  | ≥6.1 and < 7 mmol/L | 1.58 | 1.28-1.96 | <0.001 |
|  |  | ≥7 mmol/L | 2.11 | 1.79-2.49 | <0.001 |

Adjusted for age, male, GCS score, medical history, LDL-c, total cholesterol, triglyceride, HbA1c, systolic blood pressure and diastolic blood pressure.

Supplemental.Table.3 Subgroup analyses (HbA1c≥7 vs. <7) of in–hospital outcomes

|  | In−hospital outcomes | FBG level | OR | 95% CI | P value |
| --- | --- | --- | --- | --- | --- |
| HbA1c≥7 | In−hospital mortality | 3.9–6.1 mmol/L | reference | reference | reference |
|  |  | ≥6.1 and < 7 mmol/L | 0.01 | 0-9.99 | 0.98 |
|  |  | ≥7 mmol/L | 4.60 | 0.58-36.67 | 0.15 |
|  | Evacuation of intracranial hematoma | 3.9–6.1 mmol/L | reference | reference | reference |
|  |  | ≥6.1 and < 7 mmol/L | 2.72 | 1.07-6.90 | 0.04 |
|  |  | ≥7 mmol/L | 2.88 | 1.44-5.74 | 0.003 |
| HbA1c<7 | In−hospital mortality | 3.9–6.1 mmol/L | reference | reference | reference |
|  |  | ≥6.1 and < 7 mmol/L | 1.29 | 0.73-2.28 | 0.38 |
|  |  | ≥7 mmol/L | 2.11 | 1.42-3.12 | <0.001 |
|  | Evacuation of intracranial hematoma | 3.9–6.1 mmol/L | reference | reference | reference |
|  |  | ≥6.1 and < 7 mmol/L | 1.48 | 1.19-1.84 | <0.001 |
|  |  | ≥7 mmol/L | 2.01 | 1.69-2.38 | <0.001 |

Adjusted for age, male, GCS score, medical history, LDL-c, total cholesterol, triglyceride, HbA1c, systolic blood pressure and diastolic blood pressure.
